# Risk-Exposure Density and Mileage Bias in Crash Risk for Older Drivers

**DOI:** 10.1093/aje/kwx220

**Published:** 2017-06-09

**Authors:** Jonathan J Rolison, Salissou Moutari

**Affiliations:** 1 Department of Psychology, University of Essex, Colchester, United Kingdom; 2 School of Mathematics and Physics, Queen’s University Belfast, Belfast, United Kingdom

**Keywords:** crash risk, mileage bias, older drivers, risk exposure, road safety, young drivers

## Abstract

Crash rates per mile indicate a high risk of vehicle crash in older drivers. A reliance on mileage alone may underestimate the risk exposure of older drivers because they tend to avoid highways and travel more on nonfreeways (e.g., urban roads), which present greater hazards. We introduce risk-exposure density as an index of exposure that incorporates mileage, frequency of travel, and travel duration. Population-wide driver fatalities in the United States during 2002–2012 were assessed according to driver age range (in years: 16–20, 21–29, 30–39, 40–49, 50–59, 60–69, ≥70) and sex. Mileage, frequency, and duration of travel per person were used to assess risk exposure. Mileage-based fatal crash risk increased greatly among male (relative risk (RR) = 1.73; 95% CI: 1.62, 1.83) and female (RR = 2.08; 95% CI: 1.97, 2.19) drivers from ages 60–69 years to ages ≥70 years. Adjusting for their density of risk exposure, fatal crash risk increased only slightly from ages 60–69 years to ages ≥70 years among male (RR = 1.09; 95% CI: 1.03, 1.15) and female (RR = 1.22; 95% CI: 1.16, 1.29) drivers. While ubiquitous in epidemiologic research, mileage-based assessments can produce misleading accounts of driver risk. Risk-exposure density incorporates multiple components of travel and reduces bias caused by any single indicator of risk exposure.

Each year, motor vehicle collisions cause more than 1.2 million deaths (1), compromising the health and well-being of injury survivors and the families of injury victims (2). They also draw heavily on public funds through the burden they place on medical care and emergency services and through loss of productivity (3). Tighter legislation and public awareness campaigns can reduce collisions, but reliable risk indices are needed in order to target the drivers who are at greatest risk.

How should we assess driver risk? A traditional method has been to calculate crash rates per unit of travel (e.g., annual mileage (4–6)). More travel is believed to come with greater exposure to risk. Crash rates are intended to control for differences in risk exposure for group comparison in crash risk. This traditional method has led to reports of high crash risk among young and elderly drivers (5, 7–10), focusing road safety campaigns and legislation on the youngest and oldest drivers (10–12). Graduated licensing systems restrict the travel of the youngest drivers, and in many countries elderly drivers must apply regularly for renewal of a driver’s license (12, 13).

However, crash rates are not independent of travel patterns (14). Drivers who have high annual mileage tend to have a lower crash rate than that of lower-mileage drivers (15–18). This “low-mileage bias” may help explain high apparent crash rates of older drivers. Langford et al. (18) reported that the crash rate of low-mileage drivers was 6 times the crash rate of high-mileage drivers. Among driver groups with medium to high annual mileage, the crash rate of older drivers was no greater than that of drivers in other age ranges. The crash rate was higher in older age groups only among low-mileage drivers.

One prominent explanation for the low-mileage bias is that low-mileage groups contain a high proportion of impaired older drivers (17–19). Visual impairment (20) and mild cognitive impairment (21) in older drivers are associated with poorer driving ability and increased risk of crash involvement. Older drivers with visual or cognitive impairment tend to report driving less than unimpaired older drivers (22, 23). Yet Langford et al. (18) reported that crash rates were higher for all driver age ranges in the low-mileage group compared with medium- and high-mileage groups. Visual or cognitive driver impairment in older age would fail to explain a general tendency for higher apparent crash risk in low-mileage groups.

Another possibility is that drivers who have high annual mileage accumulate more miles on freeways and rural roads, whereas low-mileage drivers travel more on other road networks, such as urban networks (15, 16, 19). Urban environments present greater hazards to drivers due to their higher number of points of potential conflict (e.g., intersections, stops in traffic flow) per distance traveled (16, 19). Low-mileage drivers conduct more of their travel in urban areas than do high-mileage drivers (24). Greater exposure to more hazardous driving conditions on urban roads may explain the higher crash rates of low-mileage drivers (16). This possibility would also explain why crash rates are higher in low-mileage groups across all driver age ranges (18). Counterintuitively, low-mileage drivers may actually have a higher exposure to risk than high-mileage drivers, if a greater amount of their travel is conducted on urban roads.

Two assumptions can be made about the travel pattern of drivers who frequently travel on urban road networks. First, they should on average travel shorter distances per trip, because fewer miles per trip are accumulated on nonfreeways than on freeways and rural roads (25). Thus, drivers who more regularly use nonfreeways should have a lower travel distance per trip relative to other drivers. Second, their average travel time per mile should be higher relative to other drivers, because travel speed is typically much higher on freeways and rural roads than on urban networks (25).

On the basis of these 2 assumptions, it can be inferred that the drivers who are most exposed to risk—by driving on nonfreeways (e.g., urban roads)—should have both a low average travel distance per trip and a high average travel time per mile. In other words, the risk-exposure density of a driver group, *i*, is equal to annual travel time divided by annual travel distance (i.e., mileage), multiplied by annual travel frequency (i.e., trips), such that

(1) $$\text{Risk-exposure}\;{\mathrm{density}}_{i}=\left(\frac{{\mathrm{time}}_{i}}{{\mathrm{distance}}_{i}}\right)\times {\mathrm{frequency}}_{i}.$$

In the present study, we investigated driver risk of fatal crash on the basis of age differences in density of risk exposure. We hypothesized that 1) if older drivers travel more on nonfreeways than do middle-aged drivers, then risk-exposure density should increase in older age; and 2) if greater travel on nonfreeways explains the high crash rates of older drivers, then fatal crash risk should no longer increase in older age after accounting for age differences in risk-exposure density.

## METHODS

### Data sources

Data were collected on population-wide single- and 2-car driver fatalities recorded in the United States during 2002–2012. The data were extracted for all single-car collisions in which the driver was fatally injured. For 2-car collisions, the data were extracted for each fatally injured driver. These data were provided by the US Fatal Analysis Reporting System (US Department of Transportation) and comprise all recorded vehicle collisions on public roads resulting in a driver fatality within 30 days of a collision.

Total annual travel was assessed according to driver age range (in years: 16–20, 21–29, 30–39, 40–49, 50–59, 60–69, ≥70) and sex. The US National Household Travel Survey provided the annual trip numbers, annual mileage, and annual travel duration (in minutes) for each driver age range and sex. The US National Household Travel Survey was conducted in 2001 (51,059 drivers) and 2009 (152,857 drivers), during which the travel of each respondent was recorded over a 24-hour period. For our purposes, the travel data were averaged across the 2001 and 2009 data sets.

Average travel per driver and driver numbers in each category for age range and sex were combined to estimate total travel in each driver group. Some drivers who hold a driver’s license do not actively drive, yielding biased estimates of driver numbers. To estimate the number of active drivers in each driver group, we calculated the proportion of active drivers in the US National Household Travel Survey sample by dividing the number of drivers who made at least 1 trip during the survey period by the total number of drivers in the survey. Next, we multiplied the proportion of active drivers in the survey by the estimated number of licensed drivers in each driver group. Thus, the estimated number of active drivers reflected those who are actively engaged in driving.

### Estimation of fatal crash risk

The fatal crash rate of each driver group (according to age and sex), *i*, was estimated by dividing the annual fatal crash count of each group by its risk exposure, such that

(2) $$\mathrm{Crash}\;{\mathrm{rate}}_{i}=\frac{{\mathrm{crashes}}_{i}}{{\mathrm{exposure}}_{i}}.$$

In equation 2, exposure_*i*_ was equal to driver numbers multiplied by average trips per person (trip-based fatal crash risk), average mileage per person (distance-based fatal crash risk), or average travel duration per person (time-based fatal crash risk). In our estimate of density-based fatal crash risk, driver numbers were multiplied by risk-exposure density (equation 1). Fatal crash risk was estimated annually and was rescaled by dividing the value of each driver group by the largest value across driver groups, such that rescaled fatal crash risk equaled 1 for the driver group with the highest fatal crash risk.

### Statistical analysis

Generalized linear Poisson regression with log-link modeling was conducted to assess age differences in annual travel frequency in trips per person, annual travel distance in miles per person, annual travel duration in minutes per person, and risk-exposure density. In each regression model, age group was included as a factor. Age differences in travel distance (miles) per trip and travel time (minutes) per mile were assessed by including annual trips and annual miles as offset terms in the respective regression models. Beta regression analyses were conducted to estimate the relative risks and 95% confidence intervals for age comparison in fatal crash risk.

## RESULTS

### Demographics of travel

**Figure 1.**
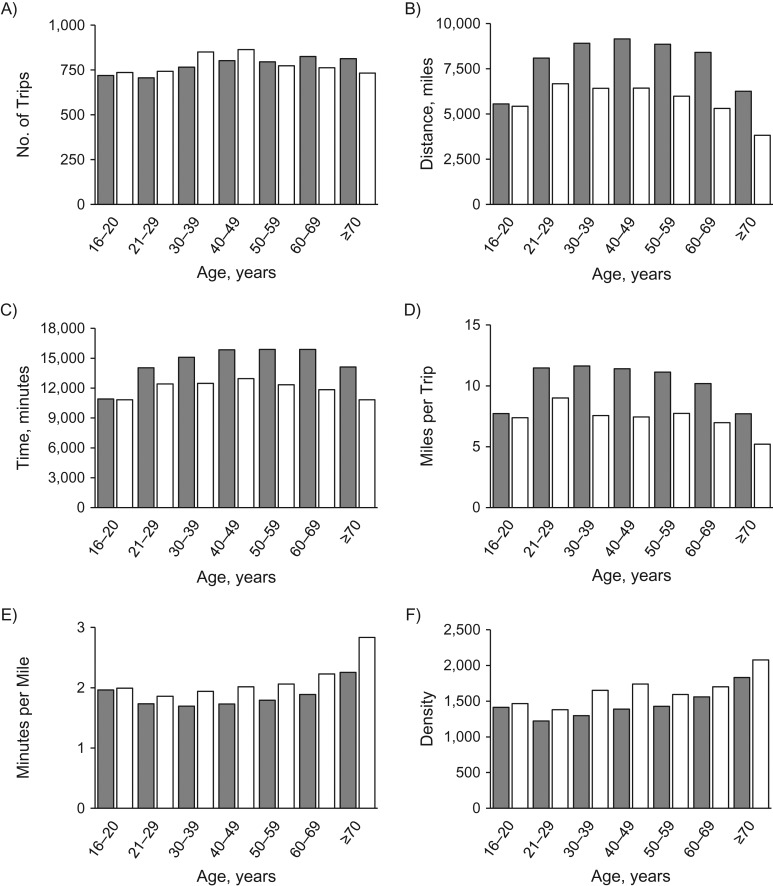
Annual travel frequency in trips (A), annual travel distance in miles (B), annual travel time in minutes (C), miles per trip (D), minutes per mile (E), and risk-exposure density (F) according to driver age range and sex, United States, 2002–2012. Gray indicates men, and white indicates women. Density equals time in minutes per distance in miles multiplied by frequency of trips.

Annual travel frequency in trips per person increased gradually from ages 16–20 years (men: 720 trips; women: 736) to ages 40–49 years (men: 802 trips; women: 864 trips) among male (relative risk (RR) = 1.11, 95% confidence interval (CI): 1.01, 1.23) and female (RR = 1.17, 95% CI: 1.06, 1.30) drivers (Figure 1A). Annual travel frequency did not decrease significantly in older age from ages 60–69 years (men: 826 trips; women: 763) to ages ≥70 years (men: 813 trips; women: 733 trips) among male (RR = 0.98, 95% CI: 0.89, 1.08) or female (RR = 0.96, 95% CI: 0.87, 1.06; Figure 1A) drivers.

Annual travel distance in miles per person increased greatly among male drivers from ages 16–20 years (5,557 miles) to ages 40–49 years (9,151 miles; RR = 1.65, 95% CI: 1.59, 1.70; Figure 1B). Annual travel distance increased to a lesser extent among female drivers from ages 16–20 years (5,431 miles) to ages 40–49 years (6,433 miles; RR = 1.18, 95% CI: 1.14, 1.23; Figure 1B). In older age, annual travel distance decreased from ages 60–69 years (men: 8,409 miles; women: 5,315 miles) to ages ≥70 years (men: 6,258 miles; women: 3,821 miles) among male (RR = 0.74, 95% CI: 0.72, 0.77) and female (RR = 0.72, 95% CI: 0.69, 0.75; Figure 1B) drivers.

Annual travel duration in minutes per person increased from ages 16–20 years (men: 10,914 minutes; women: 10,820 minutes) to ages 60–69 years among male drivers (15,882 minutes; RR = 1.46, 95% CI: 1.42, 1.49) and to ages 40–49 years among female drivers (12,961 minutes; RR = 1.20, 95% CI: 1.17, 1.23; Figure 1C). Annual travel duration decreased in older age from ages 60–69 years (men: 15,883 minutes; women: 11,846 minutes) to the age group ≥70 years (men: 14,111 minutes; women: 10,833 minutes) among male (RR = 0.89, 95% CI: 0.87, 0.91) and female (RR = 0.91, 95% CI: 0.89, 0.94; Figure 1C) drivers.

Travel distance (miles) per trip increased greatly among male drivers from ages 16–20 years (7.72 miles per trip) to ages 21–29 years (11.46 miles per trip; RR = 1.49, 95% CI: 1.44, 1.54) and increased to a lesser extent among female drivers from ages 16–20 years (7.38 miles per trip) to ages 21–29 years (9.00 miles per trip; RR = 1.22, 95% CI: 1.18, 1.26; Figure 1D). Travel distance per trip declined sharply from ages 60–69 years (men: 10.19 miles per trip; women: 6.97 miles per trip) to ages ≥70 years (men: 7.70 miles per trip; women: 5.21 miles per trip) among male (RR = 0.76, 95% CI: 0.73, 0.78) and female (RR = 0.75, 95% CI: 0.72, 0.78; Figure 1D) drivers.

Travel time (minutes) per mile reduced slightly from ages 16–20 years (men: 1.96 minutes per mile; women: 1.99 minutes per mile) to ages 21–29 years (men: 1.73 minutes per mile; women: 1.86 minutes per mile) among male (RR = 0.88, 95% CI: 0.86, 0.91) and female (RR = 0.93, 95% CI: 0.91, 0.96) drivers, and rose greatly across older age ranges, especially from ages 60–69 years (men: 1.89 minutes per mile; women: 2.23 minutes per mile) to ages ≥70 years (men: 2.26 minutes per mile; women: 2.83 minutes per mile) for male (RR = 1.19, 95% CI: 1.17, 1.22) and female (RR = 1.27, 95% CI: 1.24, 1.31; Figure 1E) drivers.

Risk-exposure density is high when travel distance per trip is low and travel time per mile is high. Accordingly, risk-exposure density decreased significantly from ages 16–20 years (men: 1,414; women: 1,467) to ages 21–29 years (men: 1,224; women: 1,380) among male drivers (RR = 0.87, 95% CI: 0.80, 0.93) but not among female drivers (RR = 0.94, 95% CI: 0.87, 1.01; Figure 1F). Risk-exposure density increased across older age ranges, especially from ages 60–69 years (men: 1,559; women: 1,700) to ages ≥70 years (men: 1,832; women: 2,078) among male (RR = 1.18, 95% CI: 1.10, 1.26) and female (RR = 1.22, 95% CI: 1.15, 1.30; Figure 1F) drivers.

### Fatal crash risk

**Figure 2.**
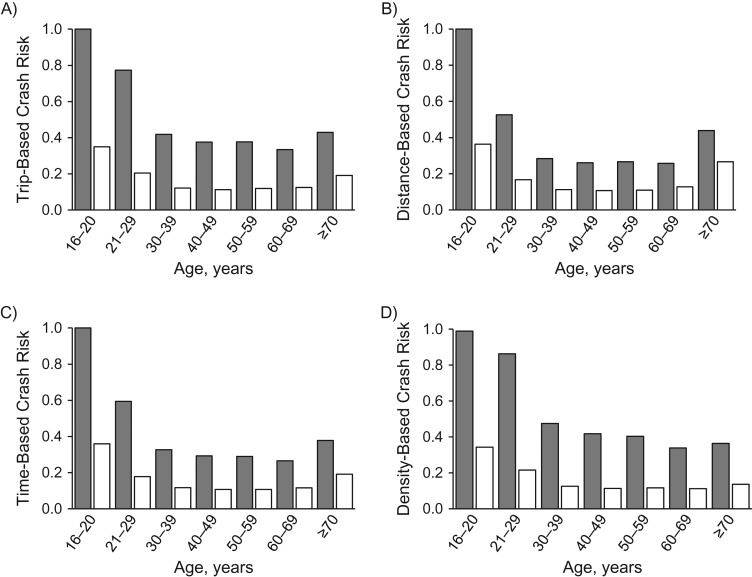
Trip-based (A), distance-based (B), time-based (C), and density-based (D) fatal crash risk by driver age range and sex, United States, 2002–2012. Gray indicates men, and white indicates women. Fatal crash risks are presented as rescaled values calculated by dividing the value of each driver group by the largest value across driver groups, where rescaled crash risk equals 1 for the driver group with the highest crash risk. Fatal crash risks are based on annual single-car and 2-car driver fatalities and annual travel and population numbers.

Trip-based fatal crash risks (Figure 2A; Table 1) were 1.25 (95% CI: 1.22, 1.29), and 1.72 (95% CI: 1.67, 1.77) times greater among male and female drivers, respectively, in the age group 16–20 years than among those in age group 21–29 years and were 2.97 (95% CI: 2.82, 3.14) and 2.83 (95% CI: 2.71, 2.94) times greater than those among age group 60–69 years. Trip-based fatal crash risk increased from ages 60–69 years to ages ≥70 years among male (RR = 1.30, 95% CI: 1.22, 1.38) and female (RR = 1.54, 95% CI: 1.45, 1.63) drivers.

**Table 1.** Trip-Based, Distance-Based, Time-Based, and Density-Based Relative Crash Risks Among Men and Women, United States, 2002–2012

Age Group, years	Trip-Based Crash Risk		Distance-Based Crash Risk		Time-Based Crash Risk		Density-Based Crash Risk	
	RR^a^	95% CI	RR^a^	95% CI	RR^a^	95% CI	RR^a^	95% CI
*Men*								
16–20	2.97	2.82, 3.14	3.90	3.67, 4.11	3.77	3.59, 3.97	2.88	2.75, 3.03
21–29	2.38	2.24, 2.52	2.07	1.95, 2.20	2.26	2.14, 2.40	2.72	2.58, 2.87
30–39	1.27	1.20, 1.34	1.13	1.06, 1.20	1.25	1.19, 1.33	1.40	1.33, 1.48
40–49	1.14	1.08, 1.21	1.04	0.98, 1.10	1.13	1.07, 1.19	1.24	1.17, 1.30
50–59	1.14	1.05, 1.22	1.05	0.98, 1.12	1.10	1.03, 1.17	1.19	1.11, 1.27
60–69	1.00	Referent	1.00	Referent	1.00	Referent	1.00	Referent
≥70	1.30	1.22, 1.38	1.73	1.62, 1.83	1.44	1.36, 1.53	1.09	1.03, 1.15
*Women*								
16–20	2.83	2.71, 2.94	2.86	2.75, 2.98	3.10	2.97, 3.24	3.07	2.95, 3.20
21–29	1.65	1.57, 1.73	1.32	1.26, 1.39	1.55	1.47, 1.63	1.93	1.84, 2.03
30–39	1.00	0.95, 1.04	0.91	0.87, 0.95	1.04	0.99, 1.08	1.14	1.09, 1.19
40–49	0.93	0.89, 0.98	0.86	0.83, 0.90	0.95	0.91, 1.00	1.03	0.98, 1.07
50–59	0.97	0.92, 1.02	0.87	0.82, 0.92	0.94	0.88, 0.99	1.04	0.98, 1.10
60–69	1.00	Referent	1.00	Referent	1.00	Referent	1.00	Referent
≥70	1.54	1.45, 1.63	2.08	1.97, 2.19	1.64	1.55, 1.73	1.22	1.16, 1.29

Abbreviations: CI, confidence interval; RR, relative risk.

^a^ Relative risk was estimated using beta regression analysis.

Distance-based fatal crash risks (Figure 2B; Table 1) were 1.89 (95% CI: 1.84, 1.93) and 2.17 (95% CI: 2.11, 2.23) times greater among male and female drivers age 16–20 years, respectively, compared with those aged 21–29 years and were 3.90 (95% CI: 3.67, 4.11) and 2.86 (95% CI: 2.75, 2.98) times greater than those among drivers aged 60–69 years. Distance-based fatal crash risk rose greatly from ages 60–69 years to ages ≥70 years among male (RR = 1.73, 95% CI: 1.62, 1.83) and female (RR = 2.08, 95% CI: 1.97, 2.19) drivers.

Time-based fatal crash risks (Figure 2C; Table 1) were 1.67 (95% CI: 1.63, 1.71) and 2.01 (95% CI: 1.95, 2.06) times greater among male and female drivers aged 16–20 years, respectively, compared with those aged 21–29 years and were 3.77 (95% CI: 3.59, 3.97) and 3.10 (95% CI: 2.97, 3.24) times greater than those among drivers in the 60–69 years age group. Time-based fatal crash risk increased in older age and was 1.44 (95% CI: 1.36, 1.53) and 1.64 (95% CI: 1.55, 1.73) times greater among male and female ≥70-year-olds, respectively, than among their counterparts aged 60–69 years.

Density-based fatal crash risks (Figure 2D; Table 1) decreased little among male drivers from ages 16–20 years to ages 21–29 years (RR = 0.94; 95% CI: 0.92, 0.96) but decreased greatly from ages 21–29 years to ages 60–69 years (RR = 2.72; 95% CI: 2.58, 2.87). Density-based fatal crash risk was 1.59 (95% CI: 1.55, 1.63) times greater among female drivers aged 16–20 years than among those aged 21–29 years and was 3.07 (95% CI: 2.95, 3.20) times greater than among those in age group 60–69 years. The density-based fatal crash risk for drivers ≥70 years of age was only slightly higher than that in age group 60–69 years among male (RR = 1.09; 95% CI: 1.03, 1.15) and female (RR = 1.22; 95% CI: 1.16, 1.29) drivers.

## DISCUSSION

Age differences in driver risk have traditionally been assessed on the basis of crash rates per unit of travel (e.g., annual mileage (4–6)). An underlying assumption of this approach is that with greater travel comes greater exposure to risk. However, older drivers regulate their travel in various ways, such as avoidance of night-time driving, poor weather, and highways (24–27). In general, more miles are accumulated on freeways and rural roads than on nonfreeways. Thus, although older drivers may have lower annual mileage, they may actually be more exposed to risk than other drivers because they conduct more of their travel on nonfreeways such as urban roads, which present more hazardous driving conditions (19).

In the present study, we introduced risk-exposure density, an index of risk exposure that incorporates annual mileage, travel frequency, and travel duration. When crash risk was based on mileage alone, risk increased greatly in older age (≥70 years) compared with age group 60–69 years (Figure 2B), reflecting findings of previous reports (6, 7). Conversely, when based on risk-exposure density, which takes account of the travel pattern, driver crash risk increased only marginally (Figure 2D). The small age-related increase in crash risk may reflect increased susceptibility to fatal injury in the elderly rather than risk of crash involvement (5). Our findings imply that driver fatality risk does not increase greatly in older age and that risk indices based on annual mileage alone can present a misleading picture of driver risk by failing to account for travel patterns.

License-renewal policies used in the United States to screen for driver impairment have an unintended outcome of discouraging unimpaired older drivers from renewing their driver’s licenses (13). Loss of driving privileges compromises mobility, which negatively affects health and well-being in older age (28, 29). Medical warnings from physicians to their patients are associated with reduction in elderly driver arrivals to emergency departments due to road traffic collisions but are also associated with an increase in visits for depression (30). Policy makers must balance a need to safeguard road users from potential harm with the benefits of maintaining mobility in older age. Our present findings imply that previous assessments of driver risk, based on annual mileage, may have exaggerated the dangers of driving in older age.

Annual mileage increased from ages 16–20 years to ages 21–29 years, which alone implies that the youngest drivers were less exposed to risk. However, risk-exposure density was higher among 16- to 20-year-olds than among 21- to 29-year-olds, owing to their lower mileage per trip and greater travel time per mile. Consequently, density-based crash risk decreased by a small amount from ages 16–20 years to ages 21–29 years in comparison with estimates of distance-based crash risk based on mileage alone. This finding suggests that previous assessments of driver risk may have exaggerated dangers faced by the youngest drivers.

High annual-mileage drivers tend to have a lower crash rate than that of drivers who travel fewer miles per year. Evidence for this “low-mileage bias” was provided by samples in which drivers involved in collisions could be stratified by their travel patterns (e.g., mileage (15, 17, 18)). In these studies, researchers were able to assess age trends in crash rates for low-, medium-, and high-mileage bands. At the national level, national travel surveys are used to adjust for demographic differences in travel (e.g., age, sex) when assessing road accident reports. These databases do not enable drivers involved in collisions to be stratified according to their personal travel patterns. Consequently, existing databases do not make it possible to assess driver crash risk per mileage category and adjust directly for a “low-mileage bias.” Our approach provides a step toward improving the reliability of national crash risk assessments by incorporating multiple components of travel to reduce bias caused by any single indicator of risk exposure.

Our study has limitations. First, our approach combines multiple components of travel to estimate driver risk exposure. This approach cannot replace existing methods of crash risk assessment in countries that do not record multiple components of travel in their national travel surveys. However, these data are recorded in the US National Household Travel Survey and thus we recommend that road-safety researchers and policy makers adopt our approach in future assessments of driver risk in the United States. Second, we proposed that travel on urban road networks is characterized by shorter distances per trip and higher travel time per mile than is travel on freeways and rural roads. Our approach does not enable us to distinguish travel on freeways and rural roads. Older adults may further differ from drivers in other age ranges in their use of rural roads versus freeways. Third, we focused our present investigation on fatally injured drivers. Motor vehicle collisions are more often fatal at high speed. However, we proposed that drivers who conduct more of their travel on high-speed freeways and rural roads are less exposed to risk of fatal crashes than drivers who avoid these road networks. One explanation is that, although traveling at high speed raises the likelihood a collision is fatal, there are far fewer collision opportunities per mile on freeways and rural roads than on urban networks, and thus many miles can be accrued on freeways and rural roads with few collision opportunities. An avenue for future research would be to compare fatal and nonfatal crash risks according to trip-, distance-, time-, and density-based indices of exposure. Finally, we did not assess driver frailty or susceptibility to physical injury. The high apparent crash risk in younger and older drivers compared with other driver age groups was lower after adjusting for their density of risk exposure. However, our approach does not enable us to unpick the contribution of crash risk and frailty to age trends in risk of fatal injury.

In conclusion, our study reveals that traditional assessments of driver risk based on annual mileage alone can provide misleading risk assessments. We incorporated annual mileage, travel frequency, and travel duration to account for travel patterns and found that driver risk does not increase greatly in older age. Risk to youngest drivers was also reduced after taking account of their travel pattern. Policy makers should be cautious when interpreting the results of mileage-based assessments of driver risk. Age trends in apparent risk depend both on the method of risk assessment used and the index of risk exposure.

## Abbreviations

CI: confidence interval
RR: relative risk
